# 3D Printed and Conventional Membranes—A Review

**DOI:** 10.3390/polym14051023

**Published:** 2022-03-03

**Authors:** Baye Gueye Thiam, Anouar El Magri, Hamid Reza Vanaei, Sébastien Vaudreuil

**Affiliations:** 1 Euromed Research Center, Euromed Polytechnic School, Euromed University of Fes, Route de Meknès (Rond point Bensouda), Fez 30000, Morocco; b.thiam@ueuromed.org (B.G.T.); s.vaudreuil@ueuromed.org (S.V.); 2 Arts et Métiers Institute of Technology, CNAM, LIFSE, HESAM University, 75013 Paris, France

**Keywords:** 3D-printed membranes, additive manufacturing, membrane process

## Abstract

Polymer membranes are central to the proper operation of several processes used in a wide range of applications. The production of these membranes relies on processes such as phase inversion, stretching, track etching, sintering, or electrospinning. A novel and competitive strategy in membrane production is the use of additive manufacturing that enables the easier manufacture of tailored membranes. To achieve the future development of better membranes, it is necessary to compare this novel production process to that of more conventional techniques, and clarify the advantages and disadvantages. This review article compares a conventional method of manufacturing polymer membranes to additive manufacturing. A review of 3D printed membranes is also done to give researchers a reference guide. Membranes from these two approaches were compared in terms of cost, materials, structures, properties, performance. and environmental impact. Results show that very few membrane materials are used as 3D-printed membranes. Such membranes showed acceptable performance, better structures, and less environmental impact compared with those of conventional membranes.

## 1. Introduction

Membrane technology, particularly polymer membranes, has multiple applications, including water treatment, electrodialysis, in batteries, and in the food and pharmaceutical industries [1,2,3]. A polymer membrane is a physical barrier separating two environments, endowed with selective permeability to certain species. In all applications, it is desirable that membranes possess high selectivity and stability, and low cost. Membrane choice depends on application type. Membranes can be of the following types: microporous, asymmetric composite thin-film, dense, or ion-exchange [1,4,5]. A microporous membrane is very similar in function to a conventional filter, where it rejects large particles (greater than 10 µm) while allowing for the smallest particles to pass [4]. For a dense membrane, permeants are transported by diffusion under the driving force of pressure, concentration, or electric potential gradient. A thin-film composite asymmetric membrane (TFC) is a microporous membrane featuring a dense thin selective layer. Ion exchange membranes can be either dense or microporous, and carry positively or negatively charged fixed ions in their polymer matrix. Their operating principle is based on the exclusion of ions of the same charge as the fixed ions of the membrane structure and the passage of ions of opposite charge.

Polymer membranes are produced using one of several approaches. Common approaches include phase inversion, stretching, track etching, sintering, electrospinning, and surface coatings of a support [1,4]. Manufacturing methods play an important role in membrane technology and its applications. Not only can membrane performance be significantly affected, but also their cost. Commercial activities and urgent needs have led to a rapid increase in membrane R&D to optimize performance, cost, and durability. Although conventional methods offer efficient membranes, the precise control of preparation parameters remains problematic. To overcome these challenges, some researchers have been adopting the additive manufacturing (AM) of membranes. AM, also called 3D printing (3DP), is considered to be a possible approach to produce custom membranes with more manufacturing control than any other method of membrane manufacturing available today [5]. Membrane 3DP has thus attracted much interest, with many research and development studies on 3D-printed membranes. Review articles attempted to provide specific discussions in this regard [5,6,7], focusing only on the discussion of 3D printing technologies [5] and their water-related applications [6,7]. However, the difference between 3D-printed membranes and conventional membranes has not been studied. Some questions remain to be clarified. Do 3D-printable materials include common materials used for membranes? Do 3D-printed membranes offer the required properties to compete with conventional membranes? Do these 3DP membranes have lower cost and environmental impact than those of conventional membranes? All these questions can lead to many thoughts about conventional and additive membranes. These are some of the topics that this article attempts to elucidate while highlighting differences between conventionally and 3D-fabricated membranes. This comparison is inevitable to evaluate the potential of 3D membranes compared to membranes produced with methods that had undergone decades of optimization. Recent developments in AM membrane production is also summarized to highlight the current research areas. This paper briefly overviews conventional and 3DP membrane fabrication methods, followed by a critical review of 3DP membranes compared to conventionally produced membranes. Prospects for developing high-performance polymer membranes highlight the potential of such manufacturing techniques.

## 2. Membrane Manufacturing Techniques

When developing high-performance membranes, researchers focus much more on materials, while paying little attention to the used manufacturing processes. These processes, however, significantly affect membrane characteristics. This section presents conventional membrane production methods and 3D printing methods.

### 2.1. Conventional Methods

**Figure 1:**
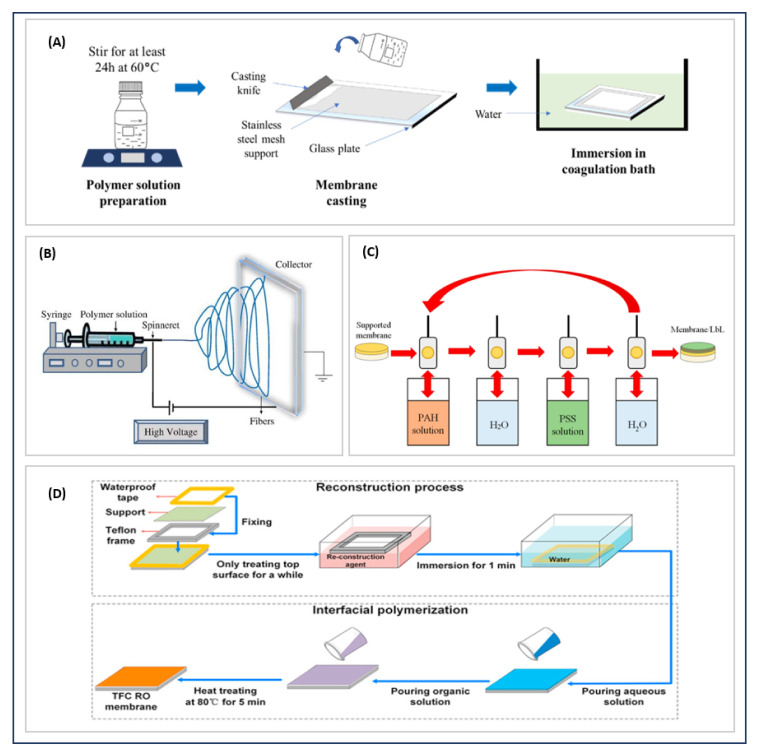
Schematic illustrations of membrane techniques. (**A**) Phase inversion [9] Reproduced from Doyan, A.; Leong, C.L.; Bilad, M.R.; Kurnia, K.A.; Susilawati, S.; Prayogi, S.; Narkkun, T.; Faungnawakij, K. Cigarette Butt Waste as Material for Phase Inverted Membrane Fabrication Used for Oil/Water Emulsion Separation. Polymers; published by MDPI, 2021. (**B**) Electrospinning [8] Reproduced from Tan, X. and Rodrigue, D., A Review on Porous Polymeric Membrane Preparation. Part I: Production Techniques with Polysulfone and Poly (Vinylidene Fluoride) Polymers; published by MDPI, 2019. (**C**) Layer by layer [10] Reproduced from Dmitrenko, M.; Kuzminova, A.; Zolotarev, A.; Ermakov, S.; Roizard, D.; Penkova, A. Enhanced Pervaporation Properties of PVA-Based Membranes Modified with Polyelectrolytes. Application to IPA Dehydration, Polymers; published by MDPI, 2021. (**D**) TFC manufacturing [11] Reproduced with permission from Shi, M.; Wang, Z.; Zhao, S.; Wang, J.; Wang, S. A Support Surface Pore Structure Re-Construction Method to Enhance the Flux of TFC RO Membrane; published by Journal of Membrane Science: published by Elsevier, 2017.

Conventional manufacturing methods are based on phase-inversion techniques, stretching, track etching, sintering, electrospinning, and layer by layer (Figure 1). Phase inversion, being a simple and fast method, is the most widely used for manufacturing membranes in which different kinds of polymers can be used for different applications. In such an approach, a polymer is first dissolved in a solvent to form a more or less viscous solution. This solution is then spread onto a glass plate and solidified [8,9]. This solidification can occur either through thermally or nonsolvent-induced phase separation.

Another approach to produce porous membranes is by stretching dense extruded films [4,12]. Stretching a dense film perpendicularly to its extrusion direction creates small breaks that result in pore formation. The stretching technique is generally used to prepare microfiltration (MF), ultrafiltration (UF), and membrane-distillation (MD) membranes, and is preferred for highly crystalline polymers [13].

Track etching is also a technique to fabricate porous membranes for various applications including filtration and cell culture [14]. Track etching instead relies on the irradiation of the dense film perpendicularly to the surface [4,14]. The radiation-damaged material is then removed by postprocessing to create straight cylindrical pores. It is an expensive technique due to the use of high-energy radiation [15]. The most commonly used materials for track etched membranes are polyethylene naphthalate (PET), polypropylene (PP), and polycarbonate (PC) [13].

Membranes can also be produced by sintering powders of polymeric materials. Compressing and heating particles slightly below their melting temperature induce bonding [8,15], with spaces between the sintered particles becoming pores. Sintering is mainly used for the preparation of microfiltration membranes. The used polymers must have excellent resistance to chemicals and high temperatures [15].

Membranes are also produced from polymer nanofibers obtained through electrospinning. Polymers such as polyvinylidene fluoride (PVDF), polyacrylonitrile (PAN), or polystyrene (PS) are electrospinable. In the process, a viscoelastic polymer solution is loaded into a syringe placed at an optimal distance from a target (or collector). A strong electrical voltage is applied between syringe and manifold to stretch droplets from the syringe tip. It generates jets of nanofibers which then settle on the collector to form an electrospun membrane [1,8], which can be used for filtration and MD processes [13].

Support coatings are conventional methods for the surface treatment of membranes. For example, the fabrication of a TFC membrane relies on interfacial polymerization. In the process, an aqueous polyamine solution is first deposited on a microporous support; then, this amine-loaded support is immersed in a diacid chloride solution. The amine and acid chloride react at the interface between the two solutions to form an extremely thin and densely cross-linked membrane layer [11,16]. Membrane surfaces can also be modified by a layer-by-layer (LBL) process where electrostatic interaction between charged surfaces are exploited through a simple immersion process. LBL can also be used to fabricate multilayer thin films [10].

### 2.2. Additive Manufacturing Method

Additive manufacturing is a layer-by-layer manufacturing process capable of easily building complex, real custom objects. Various 3D printing techniques are available such as stereolithography, digital light processing (DLP), fused deposition modeling (FDM), multijet printing (MJP), and selective laser sintering (SLS) [17,18]. All these processes work on the same basic concept to produce the final object. The whole process begins with a computer-aided design (CAD) model, which is then converted into the stereolithography format (STL). The obtained 3D file is then preprocessed by specific software, where process parameters such as 3D part orientation into the build volume and slicing parameters are defined. The information is then sent to the 3D printer that carries out layer-by-layer manufacturing.

**Figure 2:**
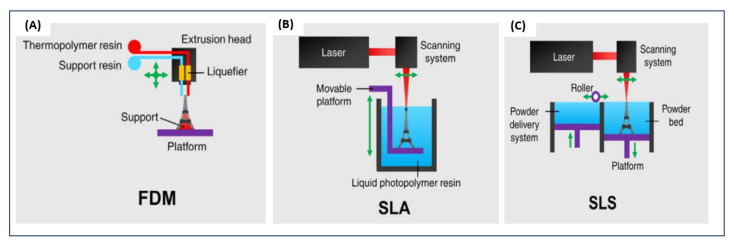
Schematic illustrations of 3D printing technologies. (**A**) FDM printing. (**B**) SLA printing. (**C**) SLS printing. [5]. Adapted from Low, Z.-X.; Chua, Y.T.; Ray, B.M.; Mattia, D.; Metcalfe, I.S.; Patterson, D.A. Perspective on 3D Printing of Separation Membranes and Comparison to Related Unconventional Fabrication Techniques, Journal of Membrane Science; Published by Elsevier, 2017.

The FDM 3D printing process (or fused filament fabrication (FFF)) consists of filament extrusion that is deposited layer by layer through a printing nozzle (Figure 2A) [19]. This deposit is produced according to the X, Y and Z coordinates of the 3D model to be printed.

Stereolithography (SLA) consists of solidifying a photosensitive liquid resin layer by layer using an ultraviolet (UV) laser beam [18,20]. As shown in Figure 2B, the build platform is initially positioned in the tank with the photopolymer resin, one layer height away from the build window. The laser beam follows a predetermined path based on the cross-section of the 3D model. After one layer is hardened, the build platform is then raised to expose a new layer of liquid polymer. The laser again traces the cross section of the object, which instantly sticks to the hardened part. A digital light-processing (DLP) projector can replace the UV laser to achieve resin hardening, enabling a cost reduction system and faster processing. However, this results in reduced XY resolution.

SLS relies on a powerful laser beam to fuse powder at very precise points of the 3D file [17,20] (Figure 2C). A new layer of fine powder is then spread before fusing the laser onto the previous layer.

## 3. Comparison of Conventional and 3DP Membranes

A comparison of 3DP polymer membranes with conventional membranes relies on available information from the literature, using a common basis. It includes material, structure, properties, performance, and cost. For example, the cost of a 3DP membrane is compared with the cost of a conventional membrane in the same application. Only some values of 3DP membrane properties were compared with those of conventional membranes due to the lack of available data for some 3DP membranes.

### 3.1. Membrane Materials

For material comparison, only the base polymer of the membrane is considered, as production of 3D membranes is usually carried out in the form of a composite membrane, i.e., 3D printing is used to manufacture the support, while other techniques are used to produce a selective layer. Materials are listed on the basis of reviews of conventional membranes [1,21,22,23,24,25], 3DP polymers [17,18,20,26], and reviewed articles on 3DP membranes.

Some 3D membrane materials are directly produced using common 3D printing technologies. FDM facilitates directly obtaining membranes from poro-lay [27,28], polylactic acid (PLA) [29], polylactide-co-glycolide (PLGA) [30], and polyethylene terephthalate (PET) [31]. The SLS technique is used to print polyamide 12 [32,33] and polysulfone [34] membranes, while SLA is used for diurethane dimethacrylate-co-polyethylene glycol diacrylate (DUDA-co-PEGDA) [35] and tangoplus [36] membranes. MJP can produce acrylonitrile butadiene styrene membranes (ABS-Like) [37,38]. Approaches based on solution casting printing allow for the direct production of PDMS [39], poly (vinyl alcohol) (PVA), polybenzimidazole (PBI) [40] and polyvinylidene fluoride (PVDF) [41] membranes. TFC membranes are also fabricated using 3D technology [42].

**Figure 3:**
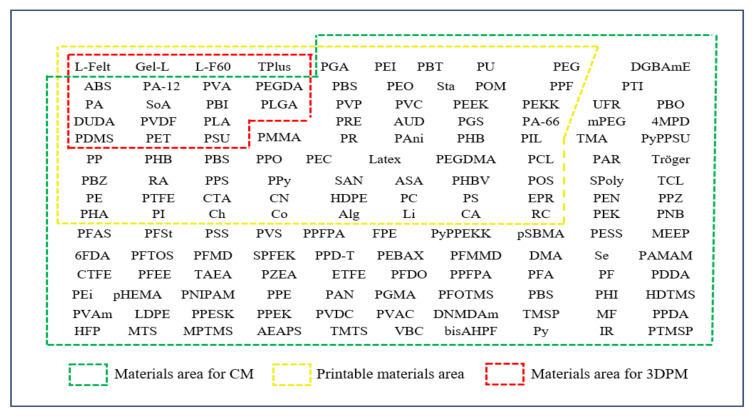
Polymers used for the manufacture of membranes: conventional membrane (CM) vs. 3D-printed membrane (3DPM) materials.

Figure 3 illustrates the materials used in conventional and 3DP membranes. Acronyms for those materials are listed and explained in the Supplementary Materials. Conventional membranes can be produced from a wide range of either natural or synthetic polymers, including vinyls, polyesters, fluorinated or chlorinated halogens, and acrylates. Very few materials are available for 3DP membranes, representing only 12% of those used in conventional membranes. The wide choice of polymers in conventional manufacturing is due to the expertise and increased development of new materials. As most polymers are soluble in solvents required to prepare cast or electrospinable solutions, this facilitates their use in phase inversion or electrospinning processes. Polymers can also be processed even without a suitable solvent, relying on sintering, track etching, or drawing processes to transform the extruded state into membranes. On the other hand, 3D printing systems are limited regarding membrane materials, as they are not compatible with all types of polymers. While printable polymers for membranes are gaining ground, the number of printed membranes remains very small. Solution casting printing can, however, allow for the printing of a wide range of currently not printable polymers [40]. If these polymers cannot be dissolved in an appropriate solvent, 3DP system development with extended printing materials is necessary.

### 3.2. Membrane Structures

According to the nature of FFF process, each deposition has its own strong influence on different aspects of the constructed parts. This issue clearly means that the final parts’ thermal, mechanical, and rheological characteristics are affected by different deposition mechanisms. There are various mechanisms of deposition based on the filling of layers, namely, counter fill, raster fill, counter, and raster fill.

**Figure 4:**
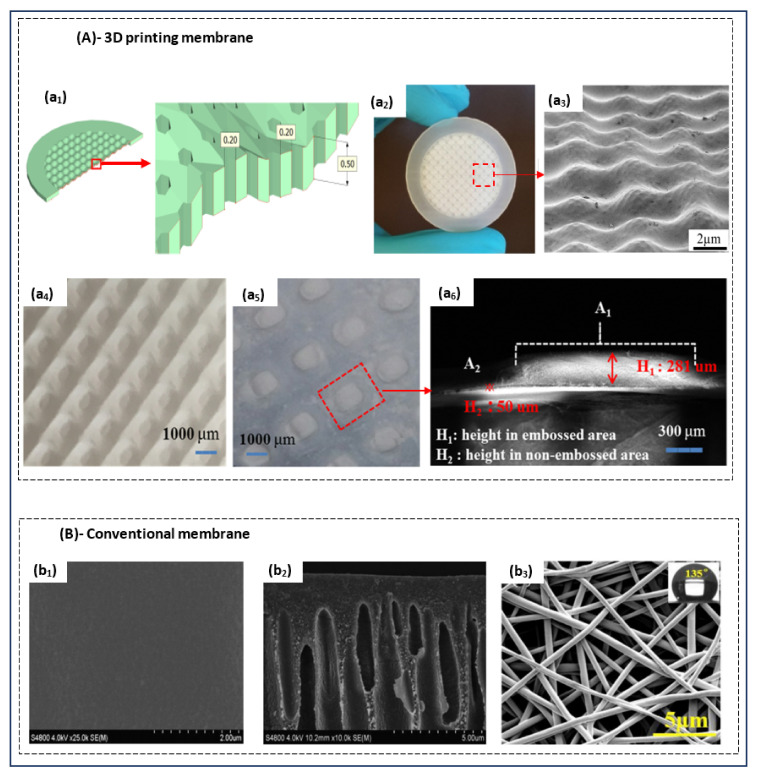
Structures of (**A**) 3D-printed and (**B**) conventional membranes. (**a1**,**a2**) 3D membrane support and its CAD, respectively; (**a3**–**a6**) 3D-printed membranes surface structures [31,37]; Reproduced with permission from Koh, E.; Lee, Y.T. Development of an Embossed Nanofiber Hemodialysis Membrane for Improving Capacity and Efficiency via 3D Printing and Electrospinning Technology, Separation and Purification Technology; published by Elsevier, 2020. Reproduced with permission from Al-Shimmery, A.; Mazinani, S.; Ji, J.; Chew, Y.M.J.; Mattia, D., 3D Printed Composite Membranes with Enhanced Anti-Fouling Behaviour, Journal of Membrane Science; published by Elsevier, 2019. (**b1**) surfaces of a conventional membrane, (**b2**) SEM micrographs of cross-sections of conventional membranes (phase inversion) [43] Reproduced with permission from Zhu, L.-J.; Liu, F.; Yu, X.-M.; Gao, A.-L.; Xue, L.-X. Surface Zwitterionization of Hemocompatible Poly(Lactic Acid) Membranes for Hemodiafiltration. Journal of Membrane Science; Elsevier 2015. (**b3**) SEM images of the surface of electrospinning membrane [44] Reproduced with permission from Zhang, Z.-M.; Gan, Z.-Q.; Bao, R.-Y.; Ke, K.; Liu, Z.-Y.; Yang, M.-B.; Yang, W. Green and Robust Superhydrophilic Electrospun Stereocomplex Polylactide Membranes: Multifunctional Oil/Water Separation and Self-Cleaning, Journal of Membrane Science; Elsevier, 2020.

The structure of a membrane influences its properties, hence the need for proper control during preparation. Structures of 3D-printed and conventional membranes are shown in Figure 4A,B, respectively. Conventional membranes generally have smooth surface morphologies (i.e., low roughness), as shown in Figure 4Bb1. Pore structure in conventional membranes, including porosity, interconnectivity, distribution, and size, is often asymmetric or unordered. For example, membranes formed by phase inversion exhibit structures characterized by their fingerlike pores under a thin layer of dense skin (Figure 4Bb2). For membranes obtained through electrospinning, a scaffold structure with disorganized but interconnected pores and low tortuosity is observed (Figure 4Bb3). This lack of uniformity in the pore structure of conventional membranes can be attributed to difficulties in controlling the preparation parameters. Although pore size can be controlled in the stretching technique, this pore formation mechanism only applies to high crystallinity polymer membranes [12]. On the other hand, 3DP membranes result from a CAD object (Figure 4Aa1), enabling the control of all parameters to achieve the desired structure. Figure 4Aa2,a3 show images of such 3DP patterned membranes. The 3DP membranes with embossed or grooved structures can easily be produced, resulting in larger surfaces than those of flat membranes. Patterned membranes are of great interest to researchers, as such membranes can exhibit improved transport performance and reduced concentration polarization while alleviating fouling [35]. The technique of 3D printing offers great manufacturing flexibility while enabling easier fabrication of complex structures than conventional methods can. The resolution limits of 3D printing are, however, limiting in membrane production. While available 3D printing methods are capable of high resolution in the z dimension, the same precision cannot be obtained for the x and y axes [7].

**Figure 5:**
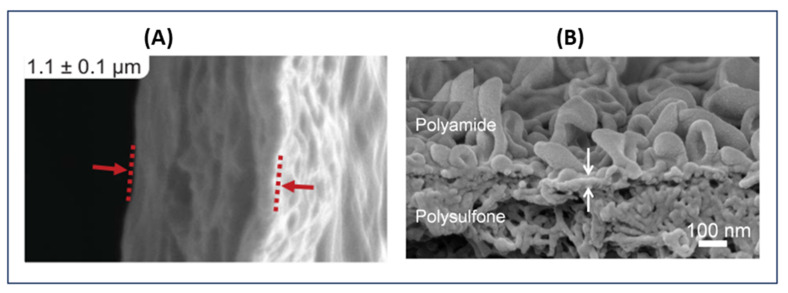
SEM images of polyamide TFC membranes with a polyamide layer: (**A**) printed [7]. Reproduced from Yanar, N.; Kallem, P.; Son, M.; Park, H.; Kang, S.; Choi, H. A New Era of Water Treatment Technologies: 3D Printing for Membranes, Journal of Industrial and Engineering Chemistry; published by Elsevier, 2020 and (**B**) conventional [47]. Reproduced with permission from Perera, D.H.N.; Song, Q.; Qiblawey, H.; Sivaniah, E. Regulating the Aqueous Phase Monomer Balance for Flux Improvement in Polyamide Thin Film Composite Membranes, Journal of Membrane Science; published by Elsevier, 2015.

If we look at TFC membranes used in desalination, the formation of the polyamide layer by interfacial polymerization is more successful for 3D printing than with the traditional method. Although conventional TFC membranes exhibit excellent permeability selectivity, their fabrication procedure is inherently limiting [42,45]. The intrinsic roughness of polyamide films has long been associated with a high fouling propensity in reverse-osmosis (RO) processes. Moreover, one cannot precisely control membrane thickness during fabrication, as the process simply self-terminates during film formation, yielding thickness of 100–200 nm [46]. The 3D printing can instead be used to deposit monomers as nanoscale droplets that forms polyamide onto a substrate. A thickness of 37 nm was achieved for 3D TFC membranes [42], meaning that the 3D membrane offers controllable roughness and independence during the in situ formation of an active polyamide film on a support. Figure 5 illustrates examples of conventional and 3DP polyamide layers.

**Figure 6:**
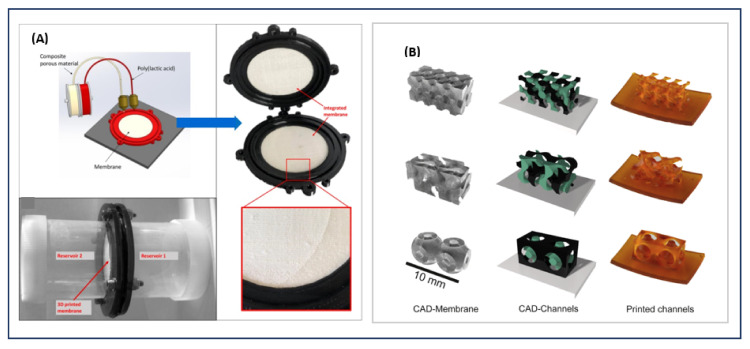
Structures of complex shapes of 3D-printed membranes. (**A**) Design of an integrated membrane device [27]. Reproduced with permission from Kalsoom, U.; Hasan, C.K.; Tedone, L.; Desire, C.; Li, F.; Breadmore, M.C.; Nesterenko, P.N.; Paull, B., Low-Cost Passive Sampling Device with Integrated Porous Membrane Produced Using Multimaterial 3D Printing; Anal. Chem., American Chemical Society, 2018. (**B**) Sheetlike triply periodic minimal-surface architecture (TPMS)-like 3D membrane [48]. Reproduced with permission from Femmer, T.; Kuehne, A.J.C.; Wessling, M. Print Your Own Membrane: Direct Rapid Prototyping of Polydimethylsiloxane, Lab Chip; published by Royal Society of Chemistry, 2014.

Most 3DP technologies do not produce membranes with the flexibility of traditional methods. However, the configuration of membranes using traditional methods is limited to simple structures (e.g., flat). This limitation can benefit the increased use of 3DP techniques where almost any complex geometric shape can be designed and produced. Examples of complex-shaped membranes are shown in Figure 6. The technology of 3D printing can create a one-print system that incorporates both the membrane and other components (Figure 6A).

### 3.3. Properties and Performance

Some of the major properties of 3D-printed membranes are given in Table 1. Thickness is a key factor in determining membrane performance. A thicker membrane generally exhibits lower permeability but higher surface resistance, thus affecting performance. The thickness of conventional membranes can reach values of 150–250 µm (for separation: e.g., water–oil), and 150 µm (for RO) [49]. The thickness of 3DP membranes is more significant, with values of 800 µm (for water–oil separation) [39] or 500 µm (water–oil separation) [37]. The thickness of a cation exchange membrane fabricated by FDM for use in microbial fuel cells reached 2000 µm [28]. It is much thicker than conventional membranes for such application, where an average thickness of 142.75 µm is found [50]. It results from the layer-by-layer operation of additive manufacturing, where the lower single layer height cannot go below 25 µm (example of SLA and DLP). The need for multiple layers to achieve structural integrity results in thicker membranes.

Pores of 3DP membranes are generally larger than those of conventional membranes for a given application. For example, 3DP membranes applied to water–oil separation have pores diameters of 370 µm [39], 200 µm [37], 250 µm [29] and 51.8 µm [34], while those in conventional membranes are generally less than 1 µm [51,53,54]. Pore size in 3DP membranes varies according to the desired structure and depends on the resolution of the used printing technology. The actual product resolution is usually lower than the nominal 3D printer resolution [5]. While most available 3D printers are not yet able to print below submicron resolution [6], two-photon polymerization (TPP) technology has achieved a resolution currently capped at ~100 nm [5]. Technologies with finer resolution are required to achieve smaller pore size without post modification.

Thickness and pores are not the only factors influencing membrane performance. Surface roughness also has positive or negative influence during application. For 3DP membranes, surface roughness depends on the 3D production technology. Conventional membranes can exhibit a rougher or smoother surface than 3DP membranes, depending on the process used. Chowdhury et al. [42] confirmed that their 3DP TFC membrane had a lower controlled roughness (~4.3 nm) than conventional TFC membranes. Reduction in roughness helps reduce the risk of membrane fouling.

Hydrophobicity or hydrophilicity are properties that could be advantageous or disadvantageous to membranes depending on the application. This depends on the used materials and/or the surface structure of the membrane. The use of a hydrophobic polymer, for example, likely results in a hydrophobic membrane. This membrane hydrophobicity is characterized by its water contact angle (WCA). Conventional membranes for separation have contact angles of 92.6° (PVDF) [12] or 21.87° (PSU) [51]. The 3DP membranes, using the same base materials and the same applications, exhibit higher contact angles at 130° (PVDF) [53] and 161° (PSU) [34]. The surface structure can also affect membrane behavior against water. 3D printing can produce superhydrophobic membranes inspired by the leaves of plants [29,54], with a structure behaving like a leaf to achieve high hydrophobicity at the surface.

Mechanical properties are also important in membrane applications. While the tensile strength of a conventional membrane used for water–oil separation can reach 1.75 MPa (PSU) [51] or 27.9 MPa (PVDF) [12], values of 17.3 (PSU) [34] and 50 MPa (PVDF) [53] were achieved for 3DP membranes. The improved mechanical properties of 3DP membranes against traditional membranes can be explained by their higher thickness. The 3DP membranes can nevertheless experience mechanical anisotropy that depends on the printing technology used and the raster orientation (layer) [6].

**Figure 7:**
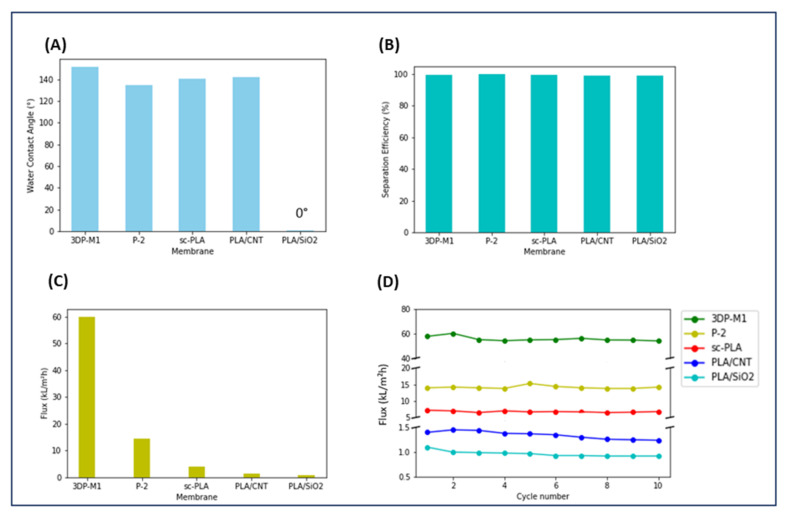
Separation performance of 3DP-M1 and conventional membranes. (**A**) Water contact angle. (**B**) Separation efficiency (n-hexane/water). (**C**) Permeation flux. (**D**) Permeation flux of n-hexane/water mixture for 10 separation cycles.

All membrane properties influence application performance. A PLA 3DP membrane decorated with polystyrene (PS) nanospheres [29], denoted 3DP-M1, was compared with conventional membranes used for water/oil separation. The performance of this membrane was compared with that of the conventional membranes of similar materials. The chosen systems include a nanofiber membrane based on PLA modified with SiO_2_ (P-2) [55], an electrospun stereocomplex PLA membrane (sc-PLA) [44], a membrane in fibrous Janus in PLA containing carbon nanotubes (PLA/CNT) [56], and another containing SiO_2_ (PLA/SiO_2_) [56]. Results of water/hexane separation efficiency and the flux of the membranes are shown in Figure 7. The separation efficiency of the membranes, including 3DP membranes, were all equal to or greater than 99%. The 3DP-M1 membrane exhibited a higher flux (60,000 LMH) than that of conventional membranes (Table 2). This flux was almost stable after 10 cycles, similar to conventional membranes. The water contact angle value for these membranes is also given in Table 2. Surface wettability has crucial influence on the oil/water separation performance of materials. A 151.7° WCA value was observed for the 3DP-M1 membrane, revealing hydrophobic behavior, while conventional membranes P-2, sc-PLA, PLA/CNT and PLA/SiO_2_ exhibited WCA of 135°, 141°, 142°, and 0°, respectively. Modifying pure PLA is thus a way to achieve a superhydrophobic surface in PLA membrane. Manufacturing membranes with lotus leaf structures can also increase hydrophobicity, feasible through a 3D printing approach.

**Figure 8:**
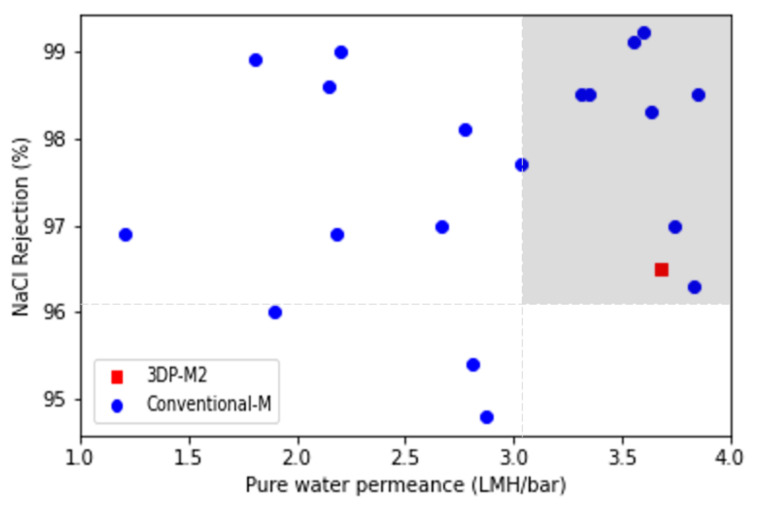
Desalination performance of printed polyamide vs. conventional membranes [46,47,57,58,59,60,61,62,63,64,65,66,67,68,69,70]. NaCl salt rejection and pure water permeance.

The water flux and salt rejection efficiency of a TFC membrane with a 3D printing deposited polyamide layer [42] were compared with those of conventional membranes using information collected from [46,47,57,58,59,60,61,62,63,64,65,66,67,68,69,70]. Figure 8 shows the performance of these membranes. The 3DP TFC membrane exhibited a >96% rejection of salt and high permeance (>3 LMH. Bar^−1^) at the same time (Figure 8, colored area). Surface roughness of ~100 nm was observed for conventional membranes [46], which is much higher than the 4.3 nm obtained for 3DP membranes. The technology of 3D printing, unlike the conventional method, can achieve a controlled polyamide layer formation, explaining the good performance achieved by 3DP TFC membrane.

A 3DP membrane offers acceptable performance in desalination and water–oil separation applications. This membrane type has also been tested in other applications with promising results. The 3DP PDMS membranes applied for gas–liquid contact showed higher CO_2_ transport in water than that of common hollow fiber membranes [48]. Philamore et al. [36] compared a conventional cation exchange membrane (CEM) of a microbial fuel cell to a 3DP membrane. The conventional CEM produced the highest power at 11.39 mW, against the 0.92 mW achieved by the Tangoplus 3DP membrane. A hemodialysis membrane fabricated via 3D printing and electrospinning technology showed a blood water removal capacity of 27% [31], while the removal of urea and NaCl during 4 h of hemodialysis reached ~17% (from 1.45 to 1.21 mg/L) and ~14% (from 0.9% to 0.8%), respectively. Isozyme clearance approached 68%.

### 3.4. Cost and Environmental Impact

Manufacturing methods affect not only membrane performance, but also the production costs. In the current circumstances, it is challenging to compare production costs of conventional and 3DP membranes, mainly due to the lack of price information in the case of 3DP membranes. Some studies nevertheless confirmed that their 3DP membrane is less expensive than conventional membranes. In the case of 3DP membranes, manufacturing costs include 3D printer purchase costs (investment) and used printing materials (consumables, operating). According to Low et al. [5,6], 3D printers are more expensive than most conventional manufacturing techniques such as solution casting, LBL, and phase inversion. This is reversed in the case of the material used during 3D printing, as Philamore et al. [36] reported significantly lower raw material costs to produce a 20 cm^2^ Tangoplus 3DP membrane compared to the equivalent area of conventional material. The Tangoplus resin used to produce a membrane costs USD 0.16, while an equivalent area of conventional membrane costs between USD 0.22 and 0.40. You et al. [28] mentioned that, while their materials are cheaper than conventional membrane material, membrane production costs (in Lay-Fomm, Gel-Lay, and Lay-Felt) were EUR 0.58–0.60 (USD 0.65–0.67). This is higher than the EUR 0.30–0.56 (USD 0.33–0.62) costs of conventional membranes [28]. Their study used 30 cm^2^ membranes, resulting in production costs of around 0.022 USD. cm^−^^2^. This is low compared to the 0.25 USD. cm^−^^2^ cost of the commonly used Nafion membrane [71]. These costs are shown in Table 3. Membrane cost would also depend on production volume. Although 3D printers are more expensive, a large production volume with inexpensive raw material results in inexpensive 3DP membranes.

One of the hazards of 3D printing processes is emissions from used materials, such as ultrafine particles (UFP) and volatile organic compound (VOC) [6]. Additive manufacturing could nevertheless be greatly significant for green environments, as waste is reduced or recycled. Large-scale conventional membrane production, on the other hand, can have potential environmental impacts because most preparations require toxic products such as N-methyl-2-pyrrolidone (NMP), N,N-dimethylformamide (DMF), or N,N-dimethylacetamide (DMAc). Although there are regulations (e.g., Registration, Evaluation, Authorization and Restriction of Chemicals (REACH)) [72] currently aimed at reducing solvent emissions and the harmful use of toxic solvents, stricter regulations require either more environmentally friendlier solvents or alternative solutions. Nonuniform manufacturing associated with these conventional methods also results in high amounts of waste [7]. As polymers used to manufacture conventional or 3D membranes are mostly derived from fossil sources, environmentally friendlier products are also needed to reduce the environmental impact.

## 4. Conclusions

In this study, conventionally prepared polymer membranes and 3D printed membranes were compared, accounting for recent developments in membrane production by additive manufacturing. Results showed differences between 3DP and conventional membranes in terms of materials, properties, performance cost, and environmental impact. This study showed that common materials for membranes are not well-adapted to additive manufacturing. This can be observed by the low number of suitable printing materials in comparison with conventional membrane materials, explainable by the inability of 3D printing technologies to use a wide range of materials. The 3DP membranes exhibited, however, a much better structure than that of conventional membranes. It can be attributed to the possibility of printing complex shapes in a controlled manner, whereas parameters are not measured or precisely controlled in conventional processes. The 3D membranes were shown to exhibit properties and performance approaching conventional membranes, and 3D printing has shown its ability of creating nature-resembling structures to improve performance. Another benefit of 3D printing is the ease in customizing membrane design to satisfy customer needs within a short turnaround time. The advantages associated with additive manufacturing could thus revolutionize the manufacture of low-cost high-performance membranes.

To achieve this, key areas must be further developed, including improvement in XY resolution and development of printers able to process a wide range of materials. The introduction of hybrid materials could be advantageous for the properties of 3DP membranes. An issue to address is the long-term stability and performance of 3D membranes, something not fully known due to the limited number of research groups working on 3D membranes. Further investigations are thus needed to demonstrate their suitability in membrane applications. Another essential area of 3DP research is the creation of a unique printing system incorporating both membrane and other components. Such development would greatly benefit membrane production. Another research direction with exciting possibilities is 4D printing, where the element of time is added to 3D printing. This enables changes in properties, function, or shape to a 3D-printed part with time [6]. Such 4D approaches could enable the production of more efficient membranes. All these eventualities, combined with larger 3D printers having very high printing speeds, can increase the potential for industrial use. Environmental considerations, including fees associated to proper waste disposal, can encourage traditional membrane manufacturing to switch to 3D printing to reduce the amount of produced waste.

## Figures and Tables

**Table 1:** Properties of 3D-printed membranes compared with conventional membranes.

Application	Membrane	Preparation Method	Thickness (µm)	Pore Size (µm)	Roughness (µm)	WCA (°)	Tensile Strength (MPa)	Reference
hemodialysis	PET(PMMA-g-PDMS)	FDM combined with Electrospinning	150	0.14	0.500	50	12	[31]
hemodialysis	PLA/PDA-g-PSBMA	Nonsolvent induced phase separation (NIPS)	35	-	-	55.1		[43]
oil–water separation	(PDMS)/SiO_2_	FDM using ink	800	370	-	160	-	[39]
oil–water separation	ABS–PES	MultiJet 3D Printing	500	200	73	83 ± 2	-	[37]
oil–water separation	PLA/polystyrène (PS)	FDM	-	250	-	151.7	-	[29]
oil–water separation	polysulfone (PSU)	SLS	355	51.8	0.135	161	17.3	[34]
ultrafiltration	PSU/Fe_3_O_4_	Electrospinning	234–241	0.07362	-	21.78	1.75	[51]
wastewater treatment	PA6	Electrospinning		0.753	-	123	0.047	[52]
filtration	PVDF	3D printing near-field electrospinning (NFES)	-	250	-	130	~50	[53]
filtration	PVDF	Melt spinning and stretching	-	0.550	3.617	92.6	27.9	[12]

**Table 2:** Comparison of 3DP-M1 performance with conventional membranes.

Membrane	WCA (°)	Flux (LMH)	Separation Efficiency (%)	Reference
3DP-M1	151.7	60,000	99.4%	[29]
P-2	135	14,379	100	[55]
sc-PLA	141	4200	99.6	[44]
PLA/CNT	142	1435	99	[56]
PLA/SiO2	0	1025	99	[56]

**Table 3:** Cost comparison of 3D and conventional membranes for microbial fuel cell.

	Cost	Reference
Material cost to produce a 3D membrane (USD/membrane)	0.16	[36]
Material cost to produce a conventional membrane (USD/membrane)	0.22–0.40	[36]
Production cost of a 3D membrane (USD cm^−2^)	0.022	[28]
Production cost of a conventional Nafion membrane (USD cm^−2^)	0.25	[71]

## Data Availability

Not applicable.

## Supplementary Materials

The following are available online at https://www.mdpi.com/article/10.3390/polym14051023/s1, List of acronyms of materials.
