# Supplementary material for: 3D Printed and Conventional Membranes—A Review
Source: Polymers (Basel). 2022 Mar 3;14(5):1023. doi: 10.3390/polym14051023 (PMC8914971; doi:10.3390/polym14051023)
Supplement: Supplementary file 1 [file polymers-14-01023-s001.zip › polymers-1599207-supplementary.pdf]

List of acronyms of materials

4MPD : 2,3,5,6-tetramethyl-1, 4-phenylenediamine
6FDA : 4,4′ -(hexafluoroisopropylidene) diphthalic anhydride
ABS : Acrylonitrile butadiene styrene
AEAPS : Aminoethylaminopropyl polydimethylsiloxane
Alg : Alginate
ASA : Acrylonitrile styrene acrylate
AUD : Aliphatic urethane diacrylate
bisAHPF : 9,9-bis(3-amino-4-hydroxyphenyl)fluorene
CA : Cellulose acetate
Ch : Chitosan
CN : Cellulose nitrate
Co : Collagen
CTA :Cellulose triacetate
CTFE : Chlorotrifluoroethylene
DGBAmE : Diethylene glycol bis(3-aminopropyl)ether
DMA : Dimethylamine
DNMDAm : 3,3′ -diamino-Nmethyldipropylamine
DUDA : Diurethane dimethacrylate
EPR : Epoxy resins
ETFE : PolyEthylene-co-Tetrafluoroethylene
FPE : Fluoropolyether
Gel-L : Gel-Lay
HDPE : High density polyethylene
HDTMS : Hexadecyltrimethoxysilane
HFP : Hexafluoropropylene
IR : Polyisoprene
LDPE : Low density polyethylene
L-F60 : Lay-Fomm 60
L-Felt : Lay-Felt
Li : Lignin
MEEP : Poly[bis(methoxy-ethoxy-ethoxy-)phosphazene]
MF : Melamine-formaldehyde
mPEG : Polyethylene glycol methyl ether
MPTMS : Mercaptopropyltrimethoxysilane
MTS : Methyltrichlorosilane
PA : Polyamide
PA-12 : Polyamide 12
PA-66 : Polyamide 66
PAMAM : Polyamidoamine
PAN : Polyacrylonitrile
PAni : Polyaniline
PAR : Polyarylate
PBI : Polybenzimidazole
PBO : Benzoxazole-phenylene
PBS : polyborosiloxane
PBS : Polybutylene succinate
PBS : Polybutylene succinate
PBT : Polybutylene terephthalate
PBZ : Polybenzoxazine
PC : Propylene carbonate
PCL : Polycaprolactone
PDDA : Polydiallydimethyl ammonium chloride
PDMS : Polydimethylsiloxane
PE : Polyethylene
PEBAX : Polyether block amide
PEC : Polyelectrolyte complex
PEEK : Polyether ether ketone
PEG : polyethylene glycol
PEGDA : Polyethylene glycol diacrylate
PEGDMA : Polyethylene glycol dimethacrylate
PEI : Polyetherimide
Pei : Polyethyleneimine
PEK : Polyether ketone
PEKK : Polyether ketone ketone
PEN : Polyethylene naphthalate
PEO : Polyethylene oxide
PESS : Polyether sulfide sulfone
PET : Polyethylene terephthalate
PF : Phenol-formaldehyde
PFA : Perfluoroalkoxy
PFAS : Perfluoroalkylsilane
PFDO : Perfluorodioxolane
PFEE : Polyfluorenyl ether
PFMD : Perfluoro-(2-methylene-1,3-dioxolane)
PFMMD : Perfluoro-(2-methylene-4-methyl-1,3-dioxolane
PFOTMS : Perfluorooctyl trimethoxysilane
PFSt : Pentafluorostyrene
PFTOS : Perfluorooctyltriethoxysilane
PGA : Polyglycolide
PGMA : polyglycidyl methacrylate
PGS: Polyglycerol sebacate
PHA : Polyhydroxyalkanoate
PHB : Polyhydroxybutyrate
PHB : Polyhydroxybutyrate
PHBV : Poly(hydroxybutyrate-co-hydroxyvalerate)
PHEMA : Polyhydroxyethyl methacrylate
PHI : polyhydroxyimide
PI : Polyimides
PIL : Polymerized ionic liquid

PLA : Polylactide
PLGA : Polylactide-co-glycolide
PMMA : Polymethyl methacrylate
PNB : Polynorbornene
PNIPAM : Poly(N-isopropylacrylamideethyl methacrylate)
POM : Polyoxymethylene
POS : Polyorganosiloxanes
PP : Polypropylene
PPDA : Pentiptycene diamine
PPD-T : Polyp-phenylene terephthalamide
PPE : Polyphenyl ether
PPEK : Polyphthalazinone ether ketone;
PPESK : Polyphthalazinone ether sulfone ketone
PPF : Poloypropylene fumarate
PPFPA : Poly(pentafluoro propyl acrylate
PPFPA : Polypentafluoropropylacrylate
PPO : Polyphenyl oxyde
PPS : Polyphenylene sulfide
PPy : Polypyrrole
PPZ : Polyphosphazene
PR : Photopolymer resin
PRE : Polymer resin E-glass
PS : Polystyrene
pSBMA : Polysulfobetaine methacrylate
PSS : Polysodium 4-styrenesulfonate
PSU : Polysulfones
PTFE : Polytetrafluoroethylene
PTI : p-Phenylene triisocyanate
PTMSP : Poly[1-(trimethylsilyl)-1- propyne]
PU : Polyurethane
PVA : Polyvinyl alcohol
PVAC : Polyvinyl acetate
PVAm : Polyvinylamine
PVC : Polyvinyl chloride
PVDC : Polyvinylidene chloride
PVDF : Polyvinylidene fluoride
PVP : Polyvinyl pyrrolidone
PVS : Polyvinyl sulfate
Py : Pyrrolidinium
PyPPEKK : Polyphthalazinone ether ketone ketone
PyPPSU : Poly(phenyl sulfone
PZEA : 2-(1-piperazinyl)ethylamine
RA : Resin ASIGA
RC : Regenerated cellulose
SAN : Acrylonitrile styrene
Se : Sericin
SoA : Sodium alginate
SPFEK : Polyfluorenyl ether ketone sulfone
SPoly : Sulfoned polymer
Sta : Starch
TAEA : Tris(2-aminoethyl)amine
TCL : Terephthaloyl chloride
TMA : Tetramethylammonium
TMSP : Trimethoxysilylpropyl
TMTS : Trimethyltrichlorosilane
TPlus :Tangoplus
UFR : Urea formaldehyde resin
VBC : Vinylbenzyl chloride
